# Enhancement of Diepoxin ζ Production by Yeast Extract and Its Fractions in Liquid Culture of *Berkleasmium*-Like Endophytic Fungus Dzf12 from *Dioscorea zingiberensis*

**DOI:** 10.3390/molecules16010847

**Published:** 2011-01-19

**Authors:** Jianglin Zhao, Bingbing Zheng, Yan Li, Tijiang Shan, Yan Mou, Shqiong Lu, Peiqin Li, Ligang Zhou

**Affiliations:** College of Agronomy and Biotechnology, China Agricultural University, Beijing 100193, China

**Keywords:** diepoxin ζ, *Berkleasmium*-like endophytic fungus Dzf12, *Dioscorea zingiberensis*, yeast extract (YE), polysaccharide fraction, non-polysaccharide fraction, enhancement

## Abstract

This study was to examine the effects of yeast extract (YE) and its fractions (YE1 and YE2) on the growth and diepoxin ζ (a spirobisnaphthalene with a diversity of bioactivities) production in liquid culture of *Berkleasmium*-like endophytic fungus Dzf12 from *Dioscorea zingiberensis*. When YE was applied to the liquid medium at 10 g/L on day 3 of culture, the diepoxin ζ production was most effectively enhanced 3.2-fold (378.70 mg/L *versus* 120.09 mg/L in control) after another 10 days culture. Feeding with 15 g/L of YE on day 9, the mycelia biomass reached 16.44 g/L, about 2.3-fold in comparison with the control (7.15 g/L). The polysaccharide fraction (YE1) was mainly responsible for stimulating diepoxin ζ accumulation, and the non-polysaccharide fraction (YE2) was mainly responsible for promoting mycelia growth. The results showed that the diepoxin ζ production in liquid culture of endophyte Dzf12 could be effectively enhanced by YE and its fractions.

## 1. Introduction

Plant endophytic fungi are an important and novel resource of natural bioactive compounds with great potential applications in agriculture, medicine and food industry [1,2,3,4]. In the past two decades, many valuable bioactive compounds with antimicrobial, insecticidal, cytotoxic and anticancer activities have been successfully discovered from endophytic fungi. These bioactive compounds could be mainly classified as alkaloids, terpenoids, steroids, quinones, isocoumarins, lignans, phenylpropanoids, phenols and lactones [5,6,7,8,9].

Spirobisnaphthalenes are a group of naphthoquinone derivatives with notable antibacterial, antifungal, antitumor, allelochemical and anti-leishmanial activities [10]. Ogishi *et al*. first reported a spirobisnaphthalene named MK 3018 from the fungus *Tetraploa aristata* [11]. After that, more than 80 spirobisnaphthalenes have been successfully obtained from nature [10]. Diepoxin ζ (also named Sch 53514, palmarumycin C13 and cladospirone bisepoxide), a spirobisnaphthalene with various bioactivities, was first isolated from the endophytic fungus LL-07F725 of a tree trunk growing in Panama [12], and later from the other fungal species such as *Nattrassia mangiferae* [13], *Coniothyrium* sp. [14], *Cladosporium* sp. [15], and *Berkleasmium*-like endophytic fungus Dzf12 [16]. It was screened to exhibit strong antitumor activity with IC_50_ values of 0.2 μM in the PLD assay [12], and 0.37 μM in an HT 1080 human fibro-sarcoma invasion assay [13], as well as strong antibacterial activity with the IC_50_ values ranging from 5.0 to 12.5 μg/mL [16]. These tremendous discoveries about diepoxin ζ have attracted many researchers’ attention. In order to speed up its applications in agriculture, medicine and food industry, the most important approach is to increase diepoxin ζ yield in fermentation culture. Many strategies (*i.e.*, medium optimization, elicitation by using polysaccharide and oligosaccharide, as well as two-phase culture) to enhance the production of bioactive compounds in either microorganism or plant cultures have been well developed so far [17,18,19,20,21,22].

Yeast extract (YE) has been widely used as the preferable nitrogen source and trace elements for the growth of microorganisms. The carbohydrate portion (main as polysaccharide) of YE has been regarded as an efficient biotic elicitor for stimulating secondary metabolites production in plant cell and tissue culture. Many valuable bioactive compounds (*i.e.*, azadirachtin, artemisinin and tanshinones) accumulation has been successfully stimulated by YE elicitors [23,24,25]. To the best of our knowledge, there were few reports about the effects of YE elicitors on secondary metabolites accumulation in fungal cell culture. The purpose of this study was to investigate the effects of YE and its two fractions (*i.e*., YE1 as polysaccharide fraction, and YE2 as non-polysaccharide fraction) on the mycelia growth and diepoxin ζ production in liquid culture of *Berkleasmium*-like endophytic fungus Dzf12 (called endophytic fungus Dzf12 or endophyte Dzf12 for short), a diepoxin ζ-producing endophytic fungus isolated from the rhizomes of a traditional Chinese medicinal plant *Dioscorea zingiberensis* C. H. Wright in our previous study [16].

## 2. Results and Discussion

### 2.1. Mycelia growth and diepoxin ζ accumulation of endophyte Dzf12 in batch culture

**Figure 1:**
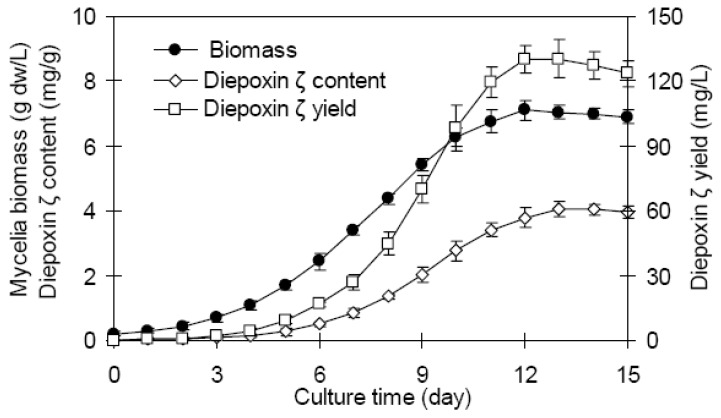
Time courses of mycelia growth and diepoxin ζ production in liquid culture of endophytic fungus Dzf12. The error bars represented standard deviations, *n* = 3.

The time courses of mycelia growth and diepoxin ζ production of endophyte Dzf12 in liquid culture with the shake-flasks are shown in Figure 1. The mycelia biomass increased slowly in the first 3 days, and increased more rapidly between day 4 and day 10 of culture, reaching the maximum biomass of 7.11 g/L around day 12. The diepoxin ζ accumulation of endophyte Dzf12 exhibited a similar time course to that of the mycelia biomass, achieving the highest content of 4.07 mg/g around day 13. Correspondingly, the maximum diepoxin ζ yield (intracellular diepoxin ζ in mycelia plus extracellular diepoxin ζ in medium) of 130.44 mg/L was obtained on day 13. The results indicated that day 13 was a suitable time for harvesting diepoxin ζ in liquid culture of endophyte Dzf12.

### 2.2. Effects of YE on mycelia growth and diepoxin ζ production of endophyte Dzf12

**Figure 2:**
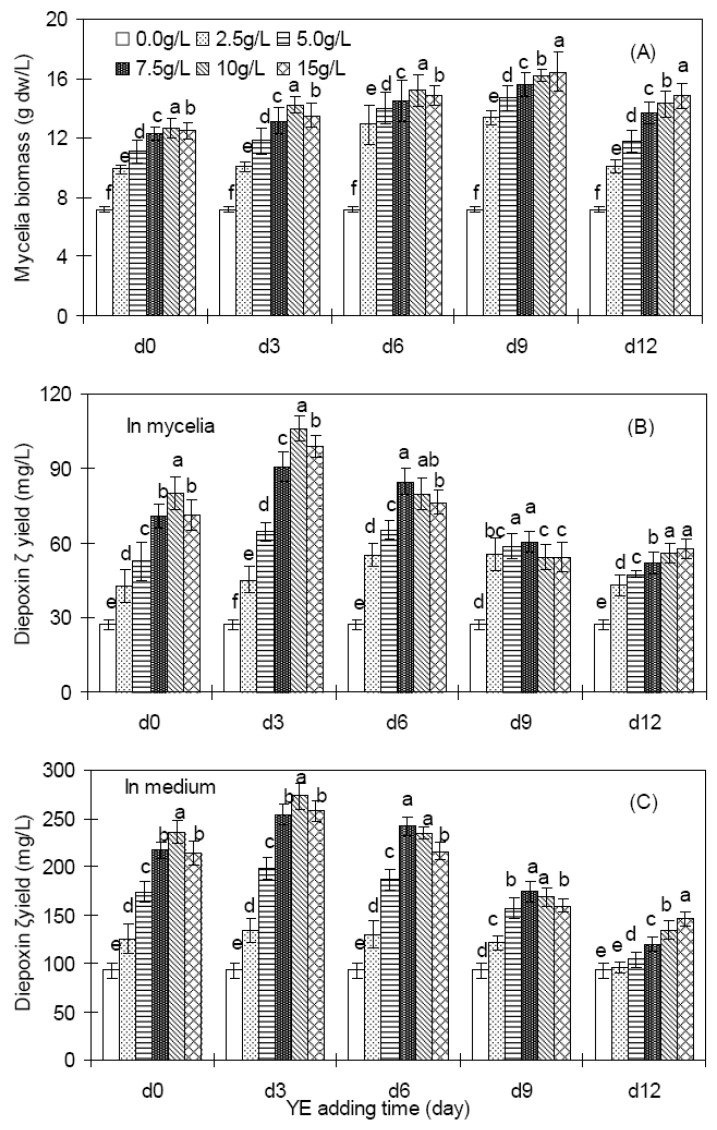
Effects of YE (0 to 15 g/L) on mycelia growth (A), intracellular diepoxin ζ production (B), and extracellular diepoxin ζ production (C) in liquid culture of endophytic fungus Dzf12. The period of culture was 13 days; The error bars represented standard deviations, *n* = 3; Different letters (*i.e*., a-f) indicated significant differences among the treatments in each YE adding time at p = 0.05 level.

Figure 2 shows the effects of YE on mycelia growth and diepoxin ζ production of endophyte Dzf12 in liquid culture, which were dependent on both YE concentration and its treatment period. As shown in Figure 2A, YE at different concentrations applied to the medium in endophyte Dzf12 liquid culture resulted in an obvious increase in biomass accumulation, most significantly at 15 g/L applied on day 9, and the mycelia biomass was increased 2.3-fold compared to that of the control (16.44 g/L *versus* 7.15 g/L). The diepoxin ζ production was also effectively enhanced by YE, most dramatically with 10 g/L of YE feeding on day 3, the intracellular diepoxin ζ of the mycelia was 106.01 mg/L, about 3.9-fold compared to that of the control 27.27 mg/L (Figure 2B), and the extracellular diepoxin ζ in the culture medium was as much as 272.69 mg/L, about 2.9-fold in comparison with the control 92.82 mg/L (Figure 2C).

**Figure 3:**
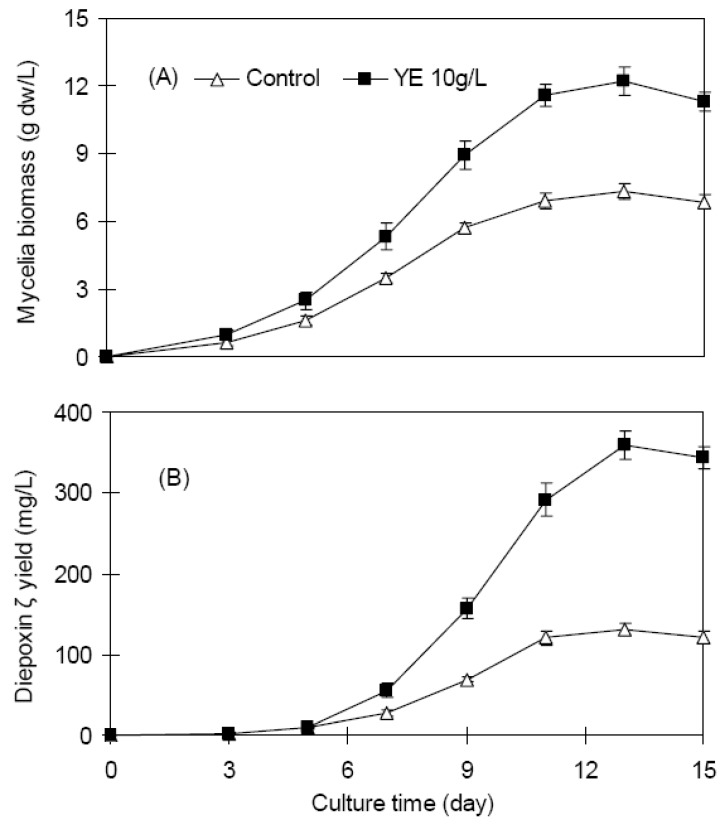
Kinetic studies of endophyte Dzf12 mycelia growth (A) and diepoxin ζ accumulation (B) after treatment with 10 g/L of YE. YE was added to the liquid medium on day 3, and the period of culture was 15 days; The error bars represented standard deviations, *n* = 3.

Figure 3 shows the time courses of endophyte Dzf12 mycelia biomass and diepoxin ζ accumulation after feeding with 10 g/L of YE. The enhancing effects of YE on mycelia biomass and diepoxin ζ production of endophyte Dzf12 could be observed on day 7, and then followed a steady increase. The highest mycelia biomass was 12.21 g/L obtained on day 13, about 1.67-fold that of the control 7.33 g/L (Figure 3A), and the total diepoxin ζ yield (the intracellular diepoxin ζ of the mycelia plus the extracellular diepoxin ζ of the culture medium) was 359.72 mg/L, about 2.8-fold compared to the control of 130.49 mg/L (Figure 3B).

### 2.3. Effects of fractions YE1 and YE2 on mycelia growth and diepoxin ζ production of endophyte Dzf12

**Figure 4:**
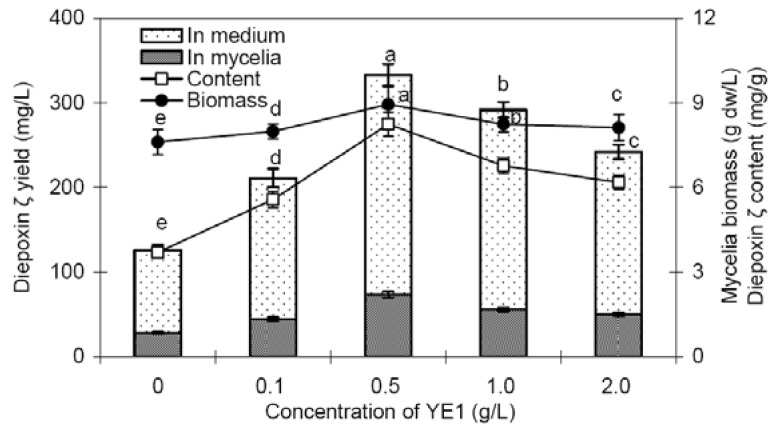
Effects of YE1 applied to the medium on day 3 on mycelia growth and diepoxin ζ production in liquid culture of endophytic fungus Dzf12. The period of culture was 13 days; The error bars represented standard deviations, *n* = 3; Different letters (*i.e*., a-e) indicated significant differences among the treatments at p = 0.05 level. Only the significant differences of the mycelia biomass and total diepoxin ζ yield were indicated.

Figure 4 shows the effects of YE1 (yeast polysaccharide) on mycelia growth and diepoxin ζ production in liquid culture of endophyte Dzf12. YE1 had only a slight enhancement effect on mycelia growth (4.9 to 17.6% increase of the mycelia biomass) but an obvious stimulatory effect on diepoxin ζ accumulation. Feeding with 0.5 g/L of YE1 on day 3, the diepoxin ζ content was as much as 8.24 mg/g, about 2.2-fold in comparison with the control of 3.70 mg/g. The intracellular diepoxin ζ yield of the mycelia was increased by 2.6-fold (73.73 mg/L *versus* 28.19 mg/L), and the extracellular diepoxin ζ yield of the culture medium was increased by 2.7-fold (259.40 mg/L *versus* 97.43 mg/L). Correspondingly, the total diepoxin ζ yield of endophyte Dzf12 liquid cultures was as much as 333.13 mg/L, about 2.7-fold that of the control 125.62 mg/L.

**Figure 5:**
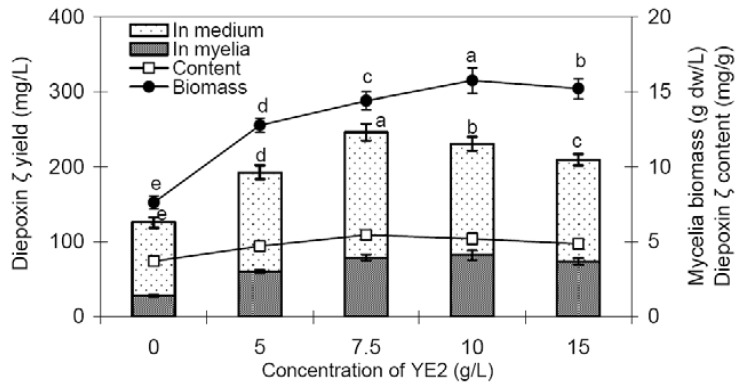
Effects of YE2 applied to the medium on day 3 on mycelia growth and diepoxin ζ production in liquid culture of endophytic fungus Dzf12. The period of culture was 13 days; The error bars for standard deviations, *n* = 3; Different letters (*i.e*., a-e) indicated significant differences among the treatments at p = 0.05 level. Only the significant differences of the mycelia biomass and total diepoxin ζ yield were indicated.

As shown in Figure 5, fraction YE2 (non-polysaccharide portion of YE) exhibited a significant promoting effect on mycelia growth. Applied 10 g/L of YE2 on day 3, the mycelia biomass reached as much as 15.75 g/L, about 2.1-fold that (7.61 g/L) of the control. YE2 also showed an obvious stimulation effect on diepoxin ζ production. Feeding with 7.5 g/L of YE2, the diepoxin ζ content of Dzf12 mycelia was 5.45 mg/g, about 1.5-fold compared to that (3.70 mg/g) of the control, and the total diepoxin ζ yield of endophyte Dzf12 liquid cultures was increased by 2.0-fold (246.20 mg/L *versus* 125.62 mg/L), although the enhancement effect was much weaker than that of YE1. The results suggested that YE1 (the polysaccharide fraction) was mainly responsible for the stimulating effect on diepoxin ζ accumulation, and the YE2 (non-polysaccharide fraction) was mainly responsible for the promoting effect on mycelia growth.

## 3. Experimental

### 3.1. Endophytic fungus and culture conditions

The endophytic fungus Dzf12 was isolated from the healthy rhizomes of the medicinal plant *Dioscorea zingiberensis* C. H. Wright (Dioscoreaceae), and identified through its morphological characteristics and internal transcribed spacer (ITS) rRNA gene sequence analysis (GenBank accession number EF543255), which gave a 91.0% sequence similarity to *Berkleasmium* sp. [16,26]. The living culture has been deposited at the China General Microbiological Culture Collection Center (CGMCC) under the number of CGMCC 2476. The stock culture of endophyte Dzf12 mycelia was maintained on potato dextrose agar (PDA) slants at 25 ºC, and in 40% glycerol at -70 ºC at the Herbarium of the College of Agronomy and Biotechnology, China Agricultural University. Liquid culture experiments were carried out in 150-mL Erlenmeyer flasks, each filled with 30 mL of the modified Sabouraud broth medium (consisted of 40 g/L glucose, 10 g/L peptone, 1.0 g/L KH_2_PO_4_, 0.5 g/L MgSO_4_·7H_2_O, 0.05 g/L FeSO_4_·7H_2_O, pH 6.5), which was favorable for the growth and diepoxin ζ production of endophyte Dzf12 in our previous investigation (data not shown), and maintained on a rotary shaker at 150 rpm and 25 ºC. The inoculum for the shake-flask culture was prepared by shaking incubation of the mycelia from the solid stock culture in potato-dextrose broth for 4 days, and 0.9 mL of mycelia broth was inoculated to each flask (3.0%, v/v).

### 3.2. Preparation and application of YE and its fractions

The yeast extract (YE) was purchased from Sigma (Y4250, St. Louis, MO, USA). YE1 was the polysaccharide fraction of YE precipitated by ethanol as described previously, and YE2 was the concentrate of the rest as the non-polysaccharide fraction [25]. Briefly, YE (20 g) was dissolved in distilled water (100 mL) and then mixed with ethanol (400 mL), and allowed to precipitate at 4 ºC for 4 days. The crude polysaccharide fraction was further purified by another round of ethanol precipitation to afford YE1 (3.32 g) which purity was determined as 95% by the anthrone test using glucose as a reference. The non-polysaccharide solution was combined and concentrated to afford YE2 (16.38 g). Both fractions YE1 and YE2 were freeze-dried and stored in a desiccator at room temperature, and the sterilized solutions were stored at 4 ºC prior to use. Elicitation treatment was carried out with YE, YE1 and YE2 as following procedures. YE was applied to the liquid medium of endophyte Dzf12 at the following five concentrations (2.5, 5.0, 7.5, 10 and 15 g/L) on days 0, 3, 6, 9 and 12 of culture, respectively. The endophyte Dzf12 liquid cultures were harvested on day 13 for measurement of the mycelia biomass and diepoxin ζ content. After the preliminary experiments, 10 g/L of YE in medium was examined to be the most effective elicitation treatment, and it was applied in the next experiments on the time courses of YE-treated mycelia growth and diepoxin ζ accumulation in endophyte Dzf12 liquid culture. Furthermore, the effects of YE1 (0.1, 0.5, 1.0 and 2.0 g/L) and YE2 (5.0, 7.5, 10 and 15 g/L) on the growth and diepoxin ζ production in liquid culture of endophyte Dzf12 were investigated.

### 3.3. Measurement of biomass and diepoxin ζ content

The mycelia of endophyte Dzf12 was separated from the liquid medium by filtration under vacuum and rinsed thoroughly with distilled water, and then dried at 50 to 55 ºC in an oven to obtain the dry weight (dw). The dried mycelia were ground into powder and then extracted with methanol/chloroform (9:1, v/v) at concentration of 10 mg mycelia per mL under sonication for 60 min. After removal of the solid, the liquid extract was evaporated to dryness and redissolved in 1 mL of methanol. For analysis of diepoxin ζ content in medium, 5 mL of the culture medium was evaporated to dryness and extracted with 5 mL of methanol/chloroform (9:1, v/v), and the liquid extraction was then evaporated to dryness and redissolved in 1 mL of methanol. The diepoxin ζ content was analyzed by high performance liquid chromatography (HPLC), consisted of Chromato Solution Light Chemstation, two LC-10ATvp pumps and a SPD-M10Avp diode-array detector (Shimadzu, Japan), and using a C_18_ column (4.6 mm × 250 mm, 5 μm, Phenomenex, Torrance, USA), methanol-H_2_O (50:50, v/v) as the mobile phase at a flow rate of 1 mL/min, and UV detection at 226 nm. The sample injection volume was 10 μL. The diepoxin ζ was detected and quantified with the standard obtained from our previous study, which was identified according to its physicochemical and spectrometric data [16].

### 3.4. Statistical analysis 

All treatments were performed in triplicate, and the results were represented by their mean values and the standard deviations (SD). The data were submitted to analysis of variance (one-way ANOVA) to detect significant differences by PROC ANOVA of SAS version 8.2.

## 4. Conclusions

This is the first report on the effects of YE and its fractions (YE1 and YE2) on the growth and diepoxin ζ production in liquid culture of *Berkleasmium*-like endophytic fungus Dzf12 associated with *D. zingiberensis*. The results showed that YE effectively enhanced both the mycelia growth and diepoxin ζ production in liquid culture of endophyte Dzf12, and the stimulation effect was concentration-dependent. Furthermore, YE1 (the polysaccharide fraction) was mainly responsible for the stimulating effect on diepoxin ζ accumulation, and YE2 (the non-polysaccharide fraction) was mainly responsible for the promoting effect on mycelia growth. Although, there are still many issues (*i.e*., the chemical composition of yeast polysaccharide, and the structure-activity relationship, the physiological responses and biochemical reactions of the fungal cells induced by the yeast elicitor) need to be further clarified and resolved. Enhancement of diepoxin ζ production by YE and its fractions in liquid culture of *Berkleasmium*-like endophytic fungus Dzf12 could be an effective strategy for large-scale production of diepoxin ζ in the future.
